# Supplementary material for: Analysis of PPARγ Signaling Activity in Psoriasis
Source: Int J Mol Sci. 2021 Aug 10;22(16):8603. doi: 10.3390/ijms22168603 (PMC8395241; doi:10.3390/ijms22168603)
Supplement: Supplementary file 1 [file ijms-22-08603-s001.zip › Supplemental materials_Analysis of PPARg signaling activity in psoriasis/Pathway models/Models images and html files/PPARG inhibits phenotypes assotiated with psoriasis/177779.html]

T-cell proliferation


# Cell Process T-cell proliferation

|  |  |
| --- | --- |
| URN | urn:agi-gocellproc:0042098 |
| Total Entities | 4 |
| Connectivity | 3609 |
| Name | T-cell proliferation |

---

|  |  |
| --- | --- |
| ChildConcepts | resting T-cell proliferation |
|  | thymocyte proliferation |
|  | alpha-beta T-cell proliferation |
|  | natural killer T cell proliferation |

---

|  |  |
| --- | --- |
| Pathway | Cortisol in Resolving Inflammation |
|  | Kynurenine Metabolites in T-Cell Apoptosis |
|  | Toll-like Receptors in Sterile Inflammation |
|  | Eicosanoids in Inflammation |
|  | Plasmacytoid Dendritic Cell in Diabetes Mellitus Type 1 |
|  | Macrophage Function in Diabetes Mellitus Type 1 |
|  | Conventional Dendritic Cell Role in Diabetes Mellitus Type 1 |
|  | Immune Sustem Activation in Hashimoto's Thyroiditis |
|  | Th1-Cell Activation in Crohn's Disease |
|  | Th17-Cell Activation in Crohn's Disease |
|  | Dendritic Cell Function in Ulcerative Colitis |
|  | T-Cell Acute Lymphoblastic Leukemia |
|  | T-Cell Genetic Rearrangements in Acute Lymphoblastic Leukemia |
|  | Antigen-Presenting Cell Role in Asthma |
|  | CD38/CD3 -> JUN/FOS/NF-kB Signaling in T-cell Proliferation |
|  | Kynurenine Metabolites Supress T-Cells in Cancer Immune Escape |
|  | Myeloid Derived Suppressor Cells in Cancer Immune Escape |
|  | IDO1 in Cancer Immune Escape |
|  | PPARG inhibits phenotypes assotiated with psoriasis |
|  | Neighbors of T-cell proliferation |
|  | 5\_Tax-mediated dysregulation of NF-kB signaling in HTLV-1 infection |
|  | Figure 4\_Diabetes mellitus Type 1: conventional dendritic cell |

---

|  |  |
| --- | --- |
| MedScan ID | 4142098 |

---

|  |  |
| --- | --- |
| Alias | T-cell proliferation |
|  | proliferation of T lymphocyte |
|  | T lymphocyte proliferation |
|  | proliferation of T-cell |
|  | proliferation of T-cells |
|  | T-cell proliferations |
|  | t cell proliferations |

---

|  |  |
| --- | --- |
| GO ID | 0042098 |

---

|  |  |
| --- | --- |
| CellType | T-cell {CellType urn:agi-ncimcelltype:C0039194} |
|  | T-cell |
|  | t cell |

---

|  |  |
| --- | --- |
| CellProcess | cell proliferation {CellProcess urn:agi-gocellproc:0008283} |

---
